# Identification of a Family of *Vibrio* Type III Secretion System Effectors That Contain a Conserved Serine/Threonine Kinase Domain

**DOI:** 10.1128/mSphere.00599-21

**Published:** 2021-08-04

**Authors:** N. Plaza, I. M. Urrutia, K. Garcia, M. K. Waldor, C. J. Blondel

**Affiliations:** a Instituto de Ciencias Biomédicas, Facultad de Ciencias de la Salud, Universidad Autónoma de Chile, Santiago, Chile; b Instituto de Ciencias Biomédicas, Facultad de Medicina y Facultad de Ciencias de la Vida, Universidad Andrés Bello, Santiago, Chile; c Division of Infectious Diseases, Brigham & Women's Hospital, Boston, Massachusetts, USA; d Department of Microbiology, Harvard Medical School, Boston, Massachusetts, USA; e Howard Hughes Medical Institute, Boston, Massachusetts, USA; UTMB

**Keywords:** *Vibrio parahaemolyticus*, NleH, VPA1328, VopG, T3SS2, T3SS, type III secretion system, foodborne pathogen

## Abstract

Vibrio parahaemolyticus is a marine Gram-negative bacterium that is a leading cause of seafood-borne gastroenteritis. Pandemic strains of V. parahaemolyticus rely on a specialized protein secretion machinery known as the type III secretion system 2 (T3SS2) to cause disease. The T3SS2 mediates the delivery of effector proteins into the cytosol of infected cells, where they subvert multiple cellular pathways. Here, we identify a new T3SS2 effector protein encoded by VPA1328 (VP_RS21530) in V. parahaemolyticus RIMD2210633. Bioinformatic analysis revealed that VPA1328 is part of a larger family of uncharacterized T3SS effector proteins with homology to the VopG effector protein in Vibrio cholerae AM-19226. These VopG-like proteins are found in many but not all T3SS2 gene clusters and are distributed among diverse *Vibrio* species, including V. parahaemolyticus, V. cholerae, V. mimicus, and V. diabolicus and also in Shewanella baltica. Structure-based prediction analyses uncovered the presence of a conserved C-terminal kinase domain in VopG orthologs, similar to the serine/threonine kinase domain found in the NleH family of T3SS effector proteins. However, in contrast to NleH effector proteins, in tissue culture-based infections, VopG did not impede host cell death or suppress interleukin 8 (IL-8) secretion, suggesting a yet undefined role for VopG during V. parahaemolyticus infection. Collectively, our work reveals that VopG effector proteins, a new family of likely serine/threonine kinases, is widely distributed in the T3SS2 effector armamentarium among marine bacteria.

**IMPORTANCE**Vibrio parahaemolyticus is the leading bacterial cause of seafood-borne gastroenteritis worldwide. The pathogen relies on a type III secretion system to deliver a variety of effector proteins into the cytosol of infected cells to subvert cellular function. In this study, we identified a novel Vibrio parahaemolyticus effector protein that is similar to the VopG effector of Vibrio cholerae. VopG-like effectors were found in diverse *Vibrio* species and contain a conserved serine/threonine kinase domain that bears similarity to the kinase domain in the enterohemorrhagic Escherichia coli (EHEC) and *Shigella* NleH effectors that manipulate host cell survival pathways and host immune responses. Together our findings identify a new family of *Vibrio* effector proteins and highlight the role of horizontal gene transfer events among marine bacteria in shaping T3SS gene clusters.

## INTRODUCTION

Vibrio parahaemolyticus is a marine Gram-negative bacterium that is the leading bacterial cause of seafood-borne gastroenteritis worldwide (1). In 1996, a new clonal V. parahaemolyticus strain of the O3:K6 serotype, now known as the pandemic clone, emerged and has been responsible for major outbreaks of gastroenteritis in diverse locations around the globe (2).

In addition to the presence of the characterized virulence factors thermostable direct hemolysin (TDH) and the *tdh*-related hemolysin (TRH), genome sequencing revealed that all V. parahaemolyticus strains encode a type III secretion system on chromosome 1 (T3SS1) (3). Furthermore, strains related to the pandemic clone harbor an evolutionarily distinct T3SS known as T3SS2 (4–6) encoded within an 80-kb V. parahaemolyticus pathogenicity island 7 (VPaI-7) on chromosome 2 (3). T3SSs are multicomponent nanomachines that enable Gram-negative bacteria to deliver proteins known as effectors directly from the bacterial cytosol into the cytosol of eukaryotic cells. Translocation of effectors into host cells enables pathogens to hijack host cell signaling, thereby manipulating a variety of host cell functions (reviewed in reference 7). Indeed, the virulence of many human, animal, and plant pathogens depends on the activity of the T3SS injectisome and the repertoire of effector proteins delivered to their respective hosts’ cells (8, 9).

Notably, most V. parahaemolyticus strains isolated from human clinical samples harbor T3SS2, and studies of animal models have shown that T3SS2 is essential for V. parahaemolyticus to colonize the intestine and to cause enteritis and diarrhea (10–12). Therefore, T3SS2 is considered a key V. parahaemolyticus virulence factor. Several T3SS2-related gene clusters have been identified in other *Vibrio* species and are referred to as T3SS2 phylotypes (T3SS2α, T3SS2β, and T3SS2γ) (13). T3SS2α include T3SS2 gene clusters related to those found in the *tdh*-positive V. parahaemolyticus pandemic strain RIMD2210633 and in Vibrio cholerae strain AM-19226. T3SS2β include T3SS2 gene clusters related to those found in V. parahaemolyticus strain TH3996 and V. cholerae strain 1587 (14). Finally, T3SS2γ include T3SS2 gene clusters related to those encoded in V. parahaemolyticus strain MAVP-Q, which has features found in the T3SS2α and T3SS2β gene clusters (15).

Nine T3SS2 effector proteins in V. parahaemolyticus have been identified thus far (VopA, VopT, VopL, VopV, VopC, VopZ, VPA1380, VopO, and VgpA) (12, 16–23). These effectors subvert several cellular pathways, including those controlling actin cytoskeleton dynamics and innate inflammatory responses (reviewed in references 13, 24, 25). These effector proteins are classified as either core or accessory T3SS2 effector proteins based on their distribution among the T3SS2 phylotypes (13). Notably, the presence of multiple uncharacterized genes in the VPaI-7 region raises the possibility that there are additional T3SS2 effector proteins yet to be identified.

In this study, we found that VPA1328, an open reading frame (ORF) in the V. parahaemolyticus VPaI-7, encodes a novel T3SS2 effector protein. VPA1328, renamed here VopG, due to its similarity to the uncharacterized V. cholerae effector VopG, is secreted in a T3SS2-dependent fashion. Comparative genomic and phylogenetic analyses revealed that VPA1328 and VopG are members of a larger family of T3SS2 effector proteins encoded within the T3SS2 clusters of vibrios outside V. parahaemolyticus and V. cholerae including Vibrio mimicus and Vibrio diabolicus and the marine bacterium Shewanella baltica. The association of *vopG* genes with insertion sequence elements in several of these clusters suggests independent horizontal gene transfer or rearrangement events in these loci. Furthermore, VopG proteins have a conserved domain that exhibits sequence and predicted structural similarity to the serine/threonine kinase domain in the well-characterized NleH family of T3SS effector proteins. These effectors have been linked to subversion of host cell survival pathways and suppression of innate immunity in infected cells (26–28). However, VopG did not block host cell death or interleukin 8 (IL-8) secretion in tissue culture-based infections, suggesting a yet undefined role for VopG during infection or functional redundancy with other T3SS2 effector proteins.

## RESULTS

### VPA1328 is a VopG homolog that is secreted and translocated into host cells by the Vibrio parahaemolyticus T3SS2.

**FIG 1:**
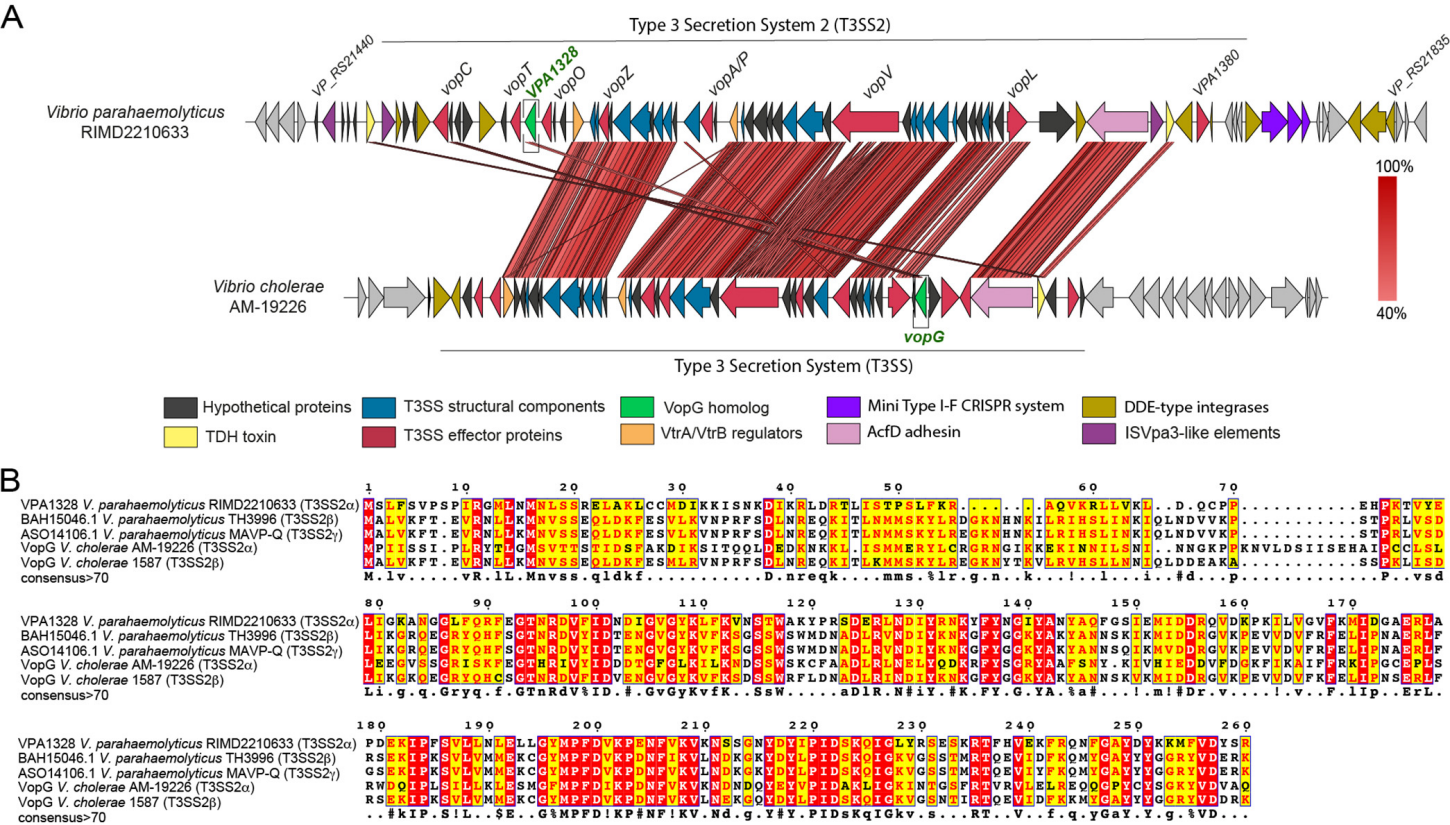
VPA1328, an ORF in the V. parahaemolyticus RIMD2210633 T3SS2 gene cluster, is similar to the VopG effector protein encoded within the V. cholerae AM-19226 T3SS2 gene cluster. (A) Schematic depiction of a comparison of the T3SS2 gene clusters in V. parahaemolyticus RIMD2210633 and V. cholerae AM-19226. BLASTn alignment was performed and visualized using Easyfig. (B) Multiple sequence alignment of VPA1328 and VopG homologs. BLASTp alignments were performed using T-Coffee Expresso and visualized by ESPript 3.0. Amino acids with a red background correspond to positions with 100% identity; amino acids with a yellow background correspond to positions with >70% identity.

We carried out BLASTp-based homology searches of the V. parahaemolyticus VPaI-7 genomic island as a way to identify candidate new T3SS2 effector proteins. This approach suggested that VPA1328 (VP_RS21530 in the latest genome annotation of strain RIMD2210633) is a putative T3SS2 effector protein (see Table S2 in the supplemental material). VPA1328 is predicted to encode a 260-amino-acid protein that shares ∼42% amino acid sequence identity with the T3SS effector protein VopG, encoded in the phylogenetically related T3SS2 in V. cholerae AM-19226 (29) (Fig. 1A and B). The function of VopG remains unknown, but it is secreted and translocated by the V. cholerae T3SS2 and contributes to host cell cytotoxicity and colonization in a mouse model of infection (29), suggesting an important role for this effector in virulence (29). Even though VPA1328 and VopG are located in different locations within their respective T3SS2 clusters, their sequence similarity and presence in phylogenetically related T3SSs suggest that VPA1328 is a VopG homolog that functions as a V. parahaemolyticus T3SS2 effector protein. Below we refer to VPA1328 as VopG.

10.1128/mSphere.00599-21.7 TABLE S2 Analysis of the VPA1328 ORF using different T3SS effector prediction software. Download Table S2, XLSX file, 0.01 MB. Copyright © 2021 Plaza et al. 2021 Plaza et al. https://creativecommons.org/licenses/by/4.0/ This content is distributed under the terms of the Creative Commons Attribution 4.0 International license.

Next, we tested whether VopG (VPA1328) is secreted and whether its secretion requires the V. parahaemolyticus T3SS2. In these experiments, V. parahaemolyticus wild-type (WT) strain RIMD2210633 and isogenic T3SS1, T3SS2, and T3SS1/T3SS2-deficient mutant strains (Δ*vscn1*, Δ*vscn2*, and Δ*vscn1* Δ*vscn2*, respectively) were grown under conditions (26) that induce expression of T3SS2 (LB 0.04% bile) (30). To detect VopG, these strains were transformed with pVPA1328-CyaA, a plasmid that harbors a translational fusion between the VPA1328 ORF (VopG) and the adenylate cyclase domain (CyaA) of plasmid pCyaA. This construct enables immunoblot detection of VopG in cell lysates and culture supernatants using anti-CyaA antibodies. A VopV-CyaA fusion (pVopV-CyaA) was included as a positive control for T3SS2-dependent secretion (19).

**FIG 2:**
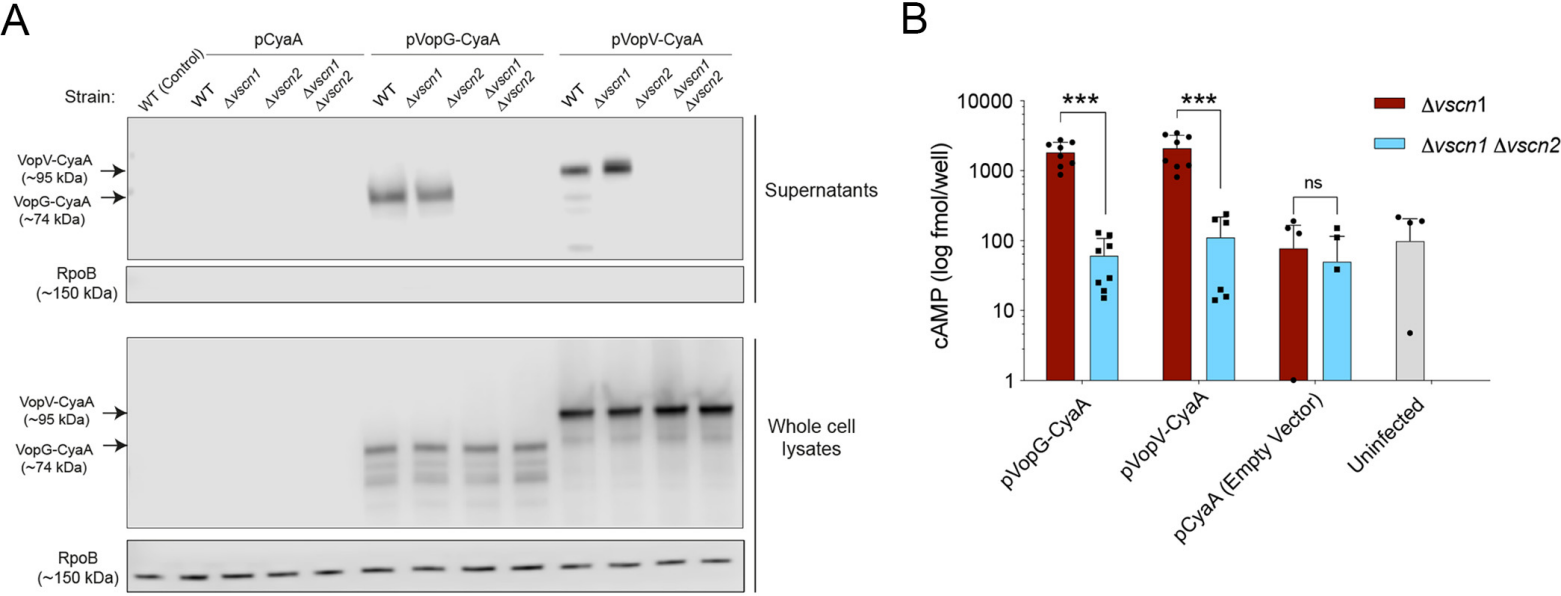
VPA1328 is secreted and translocated in a T3SS2-dependent fashion. (A) Immunoblots of culture supernatants and whole-cell lysates using anti-CyaA antibodies for CyaA-tagged VPA1328 (VopG) and VPA1357 (VopV) in wild-type (WT) (A) and isogenic Δ*vscn1*, Δ*vscn2*, and Δ*vscn1* Δ*vscn2* mutant strains. Immunoblots for RpoB were used as a control for cell lysis. (B) Translocation of VopG-CyaA and VopV-CyaA fusions was assessed via determination of intracellular cAMP levels of infected Caco-2 cells. Values are means plus standard deviations (error bars) from two independent biological replicates each with four technical replicates. Asterisks indicate significant differences (*t* test, ***, *P* < 0.001). ns, not significantly different.

A band corresponding to the predicted size of the VopG-CyaA fusion (∼74 kDa, along with some lower-molecular-weight species likely corresponding to degradation products) was observed in cell lysates from the WT strain harboring pVopG-CyaA, but not a control strain harboring the empty vector pCyaA (Fig. 2A). VopG was detected only in supernatants when the WT (pVopG-CyaA) strain was grown under T3SS2-inducing conditions (LB 0.04% bile) and not in culture supernatants in strains lacking a functional T3SS2 (Fig. 2A), suggesting that its secretion requires T3SS2 activity. Interestingly, previous transcriptomic analysis showed that expression of VPA1328 was increased by the presence of bile and controlled by VtrB, the master regulator of T3SS2 expression, suggesting that it is part of the VtrB regulon (31).

Analyses of VopG secretion from Δ*vscn1* (T3SS1-deficient) and Δ*vscn2* (T3SS2-deficient) strains strongly support the idea that VopG secretion requires T3SS2 and not T3SS1. When secretion by T3SS1 or T3SS2 or both T3SS was disabled by deletion of their respective ATPases, there was similar expression of VopG in cell lysates (Fig. 2A); however, VopG was detected only in supernatants from the strain where T3SS1 was inactivated but not when T3SS2 was inactive. An identical pattern was observed with VopV, a known T3SS2 substrate (Fig. 2A). The cytosolic RNA polymerase beta subunit (RpoB) was not detected in any of the culture supernatant samples, indicating that detection of VPA1328 in culture supernatants was not a consequence of bacterial lysis.

We also tested whether VPA1328 (VopG) is translocated into infected host cells by V. parahaemolyticus *T3SS2*. Caco-2 cells were infected with V. parahaemolyticus strains harboring the effector-CyaA reporter fusions described above, and the amount of intracellular cyclic AMP (cAMP) generated by each translocated effector was measured using an enzyme-linked immunosorbent assay (ELISA). As shown in Fig. 2B, after 1 h of infection, cAMP levels generated by VopV-CyaA (positive control) and VopG-CyaA were similar and significantly higher in cells infected with V. parahaemolyticus strains harboring a functional T3SS2 (Δ*vscn1*, T3SS2+) than in cells infected with a T3SS-deficient strain (Δ*vscn1* Δ*vscn2*, T3SS−), which exhibited background cAMP levels (Fig. 2B). Together, these observations demonstrate that VopG is secreted and translocated into host cells in a T3SS2-dependent fashion and given its similarity to the V. cholerae VopG effector, strongly support the notion that VopG is a novel V. parahaemolyticus T3SS2 effector protein.

### VopG homologs are widely distributed in vibrios harboring T3SS2 clusters.

**FIG 3:**
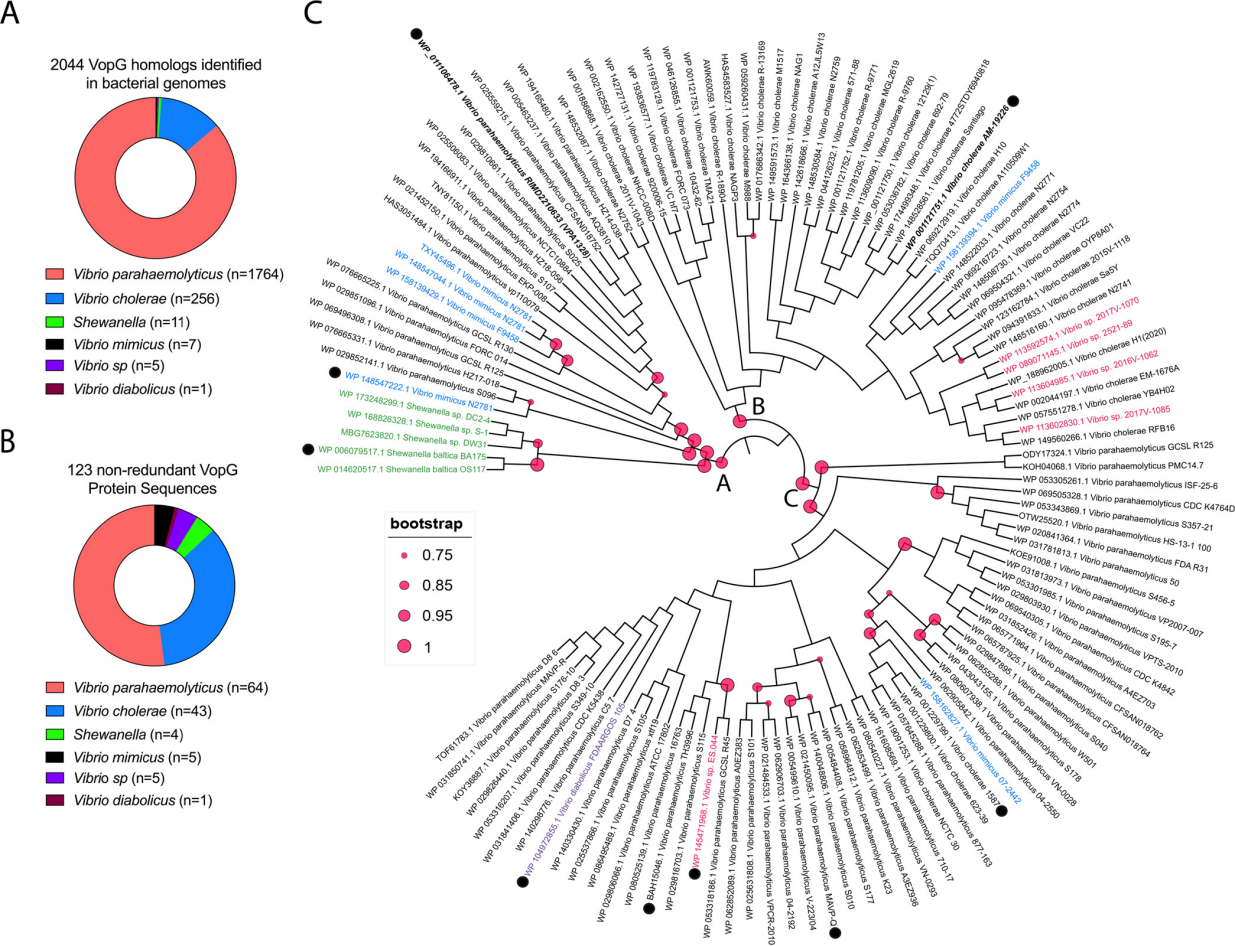
VopG homologs are widely distributed among *Vibrio* and *Shewanella* species. (A) Number of *Vibrio* sp. and *Shewanella* sp. isolates where VopG homologs were identified. (B) Distribution of the 123 nonredundant VopG protein sequences in different *Vibrio* and *Shewanella* species. (C) Phylogenetic analysis of the 123 nonredundant VopG homologs identified in this study. Phylogenetic analysis was performed with MEGA and visualized by iTOL. Distinct bacterial species are highlighted in different colors. VopG homologs used in the multiple sequence alignment of Fig. 5A are highlighted with a black dot.

The presence of a VopG homolog encoded within the T3SS2 gene cluster in V. parahaemolyticus RIMD2210633 prompted us to investigate whether additional VopG homologs are present among distinct T3SS2 phylotypes. The VPA1328 sequence was used as a query to identify potential VopG homologs by sequential BLASTn, BLASTp, and tBLASTx searches, using publicly available bacterial genome sequences. With cutoff values of 60% sequence coverage and 40% sequence identity, 2,044 candidate VopG homologs were identified, including 123 nonredundant protein sequences (Fig. 3A and B and Table S3). The majority of the VopG homologs (86%; *n* = 1,764) were encoded in V. parahaemolyticus strains and in V. cholerae strains (12.5%; *n* = 256), but homologs were also identified in V. mimicus (0.2%; *n* = 5), V. diabolicus (0.04%; *n* = 1), and *Vibrio* sp. (0.5%; *n* = 11) and in *Shewanella* strains (0.3%; *n* = 7), i.e., in most species known to harbor T3SS2 gene clusters. Interestingly, a T3SS2 gene cluster was not previously identified in *V. diabolicus*, a marine organism. However, it is important to note that not all *Vibrio* species, e.g., Vibrio anguillarum (32) which harbors T3SS2 gene clusters, carry genes that encode VopG homologs. Thus, even though VopG is widely distributed, this putative effector protein is not a universal component of the T3SS2.

10.1128/mSphere.00599-21.8 TABLE S3 List of the 2,044 total VopG protein sequences and the 123 nonredundant VopG protein sequences identified in bacterial genome databases. Download Table S3, XLSX file, 0.1 MB. Copyright © 2021 Plaza et al. 2021 Plaza et al. https://creativecommons.org/licenses/by/4.0/ This content is distributed under the terms of the Creative Commons Attribution 4.0 International license.

Next, we evaluated the sequence relatedness of VopG homologs using phylogenetic analysis of the 123 nonredundant VopG sequences. Three distinct clades (A, B, and C) of VopG proteins were identified (Fig. 3C), but no clear correlation was found between these clades and T3SS2 phylotypes. For example, the VopG homologs of V. cholerae strain AM-19226 and V. parahaemolyticus RIMD2210633 (VPA1328) clustered in different clades (B and A, respectively) despite the fact that both these T3SS2 belong to the T3SS2α phylotype. The lack of correlation between the VopG clades and T3SS2 phylotypes suggests that VopG effectors have to some extent evolved independently of the T3SS2 machinery that delivers them to host cells.

**FIG 4:**
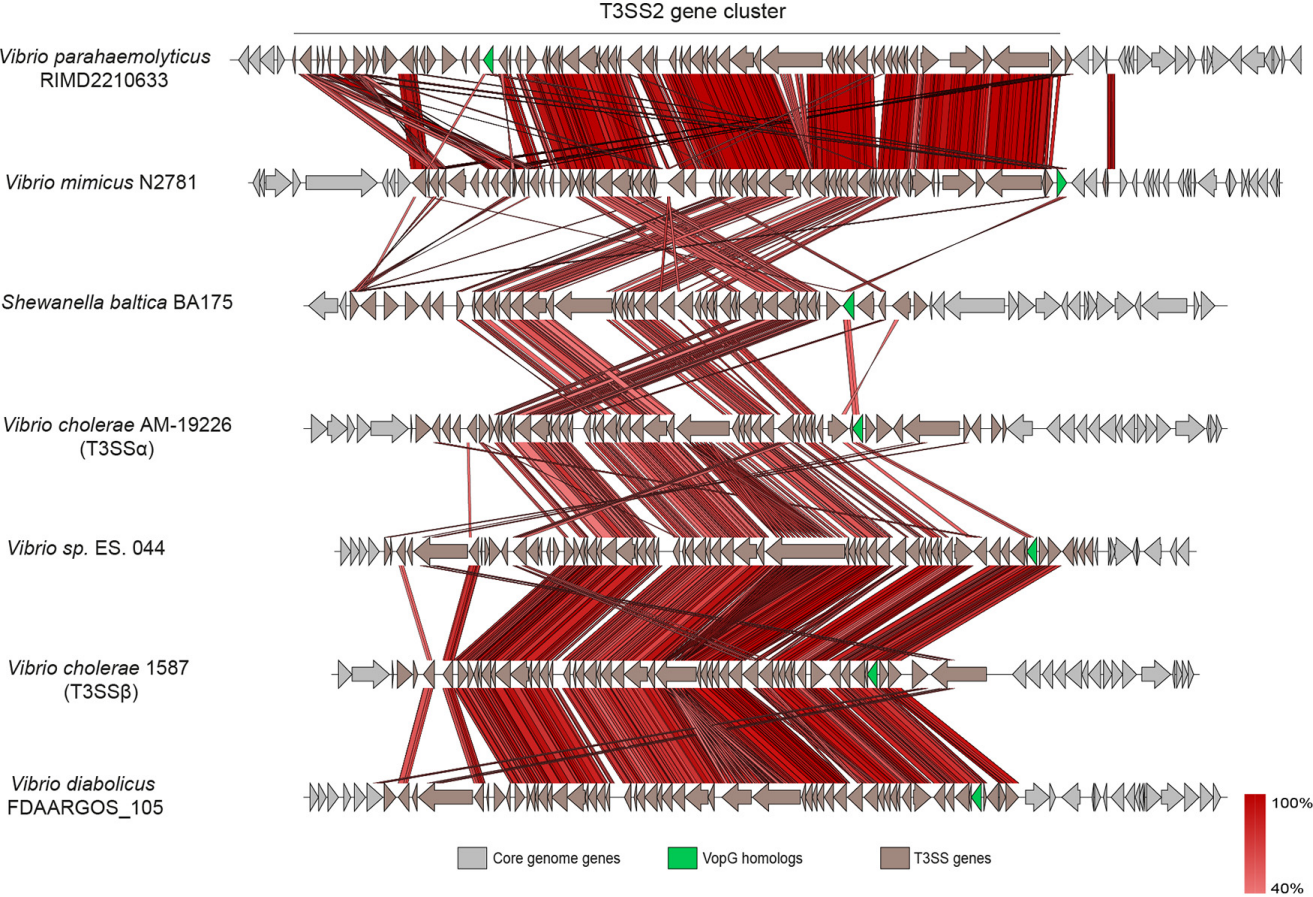
Locations of *vopG* homologs within T3SS2 gene clusters. BLASTn alignments were performed and visualized using Easyfig.

Comparative genomic analyses were carried out to gain insights into variation of the genomic contexts of *vopG* genes within different T3SS2 gene clusters. Genome sequences from representatives of each clade of the VopG phylogenetic tree, including at least one genome for each different *Vibrio* species were used for these comparisons. As shown in Fig. 4, the overall genetic structure of these T3SS2 gene clusters is highly conserved, particularly in the regions encoding structural components of the T3SS2 apparatus. In most T3SS2 gene clusters, the relative position of *vopG* was similar with the exception of V. parahaemolyticus RIMD2210633. However, the nucleotide sequences and ORFs that are adjacent to the *vopG* homologs differed in most of the seven clusters analyzed in Fig. 4. In several of these cases, *vopG* was found to be close to sequences related to insertion sequence (IS) elements. This association raises the possibility that IS elements can promote the mobility of *vopG* loci and potentially account for the variations in the genetic contexts of these loci within different T3SS2 gene clusters.

Consistent with this idea, we identified two *vopG* homologs (FORC14_RS05860 and FORC14_RS06170) encoded in the T3SS2 gene cluster of V. parahaemolyticus strain FORC014. Analysis of their respective genetic contexts revealed that one of these *vopG* genes (FORC14_RS05860) is located at the end of the T3SS gene cluster and is flanked by IS*200*-like mobile genetic elements (see Fig. S1A in the supplemental material). These elements have high sequence identity to the ISVpa3 insertion sequence. ISVpa3 is an insertion sequence located adjacent to each copy of the TDH gene in V. parahaemolyticus strain RIMD2210633 and linked in some strains to deletion of TDH (33). While the T3SS2 gene cluster of V. parahaemolyticus RIMD2210633 has three copies of these ISVpa3 elements, V. parahaemolyticus strain FORC014 has six of these elements, two of them flanking one of the *vopG* homologs at the end of the cluster (Fig. S1B). Sequence analysis showed that the two *vopG* homologs in strain FORC014 share 69% nucleotide identity (Fig. S2). Both the sequence divergence of these two *vopG* genes and the mobile genetic elements flanking FORC14_RS05860 suggest that this *vopG* homolog was independently acquired, potentially via a horizontal gene transfer event, and not a duplication of FORC14_RS06170.

10.1128/mSphere.00599-21.1 FIG S1 Genomic context of *vopG* in Shewanella baltica BA175 and V. parahaemolyticus FORC014. (A) Schematic of the *vopG* genomic context within T3SS2 gene clusters of *Shewanella* species and within the T3SS gene cluster of V. parahaemolyticus FORC014 (B). BLASTn alignment was performed and visualized using Easyfig. Download FIG S1, PDF file, 0.8 MB. Copyright © 2021 Plaza et al. 2021 Plaza et al. https://creativecommons.org/licenses/by/4.0/ This content is distributed under the terms of the Creative Commons Attribution 4.0 International license.

10.1128/mSphere.00599-21.2 FIG S2 Multiple sequence alignment of the DNA sequence of the *vopG* homologs encoded within the T3SS2 gene cluster of V. parahaemolyticus strain FORC014. BLASTn alignment was performed using T-Coffee and visualized by ESPript 3.0. Nucleotides on a red background correspond to positions with 100% identity. Download FIG S2, PDF file, 0.9 MB. Copyright © 2021 Plaza et al. 2021 Plaza et al. https://creativecommons.org/licenses/by/4.0/ This content is distributed under the terms of the Creative Commons Attribution 4.0 International license.

While the presence of a T3SS2 gene cluster in Shewanella baltica species has been inferred due to the presence of the *vscn2* gene in strain S. baltica BA175 and S. baltica OS183 (34), information regarding the distribution and genetic context of the T3SS2 gene cluster in this genus has not been reported. We found that the *Shewanella* T3SS2 is located within a genomic island inserted between the SBAL678_RS45345 and SBAL678_RS45350 ORFs of reference strain OS678 (Fig. S1B). This genomic island includes 45 ORFs. Most of these genes encode structural components of the T3SS2 apparatus. Interestingly, not every T3SS2 gene cluster identified in *Shewanella* harbors a VopG-encoding gene (Fig. S1B).

### VopG proteins have sequence and predicted structural similarity to the NleH family of serine/threonine kinases.

The amino acid sequence conservation of the 123 VopG homologs was analyzed and depicted using WebLogo. The analysis showed a particularly striking conservation in the C termini of these amino acid sequences (Fig. S3), suggesting that this region of VopG includes a functional domain. To gain clues regarding the function of VopG proteins, we used the structure-based homology tools HHpred (35) and pGenTHREADER (36). For these analyses, the amino acid sequence of V. parahaemolyticus VPA1328 was used as a representative of the VopG family of effectors. Both algorithms detected a region in the VopG C terminus with similarity to NleH effectors, e.g., HHpred analysis uncovered the presence of a region of 22 amino acids in VPA1328 (positions 190 to 212) with identity to the T3SS effector proteins NleH1 from Escherichia coli O157:H7 strain Sakai (PDB accession no. 4LRJ chain B) and OspG from Shigella flexneri 2a strain 301 (PDB accession no. 4Q5E chain A).

10.1128/mSphere.00599-21.3 FIG S3 WebLogo analysis of the multiple sequence alignment of the 123 nonredundant VopG proteins identified in this study. Download FIG S3, PDF file, 0.3 MB. Copyright © 2021 Plaza et al. 2021 Plaza et al. https://creativecommons.org/licenses/by/4.0/ This content is distributed under the terms of the Creative Commons Attribution 4.0 International license.

NleH1 and OspG are members of the NleH family of T3SS effectors (37–39). These proteins are translocated by the T3SSs of different bacterial species and act as serine/threonine kinases in host cells (38). There are six members of this family of effector proteins, including the NleH1 and NleH2 proteins of E. coli O157:H7 strain Sakai, OspG from Shigella flexneri 2a strain 301, NleH of Citrobacter rodentium strain DBS100, SboH of Salmonella bongori NCTC 12419, and YspK of Yersinia enterocolitica strain 8081 (26, 28, 40–43). The serine/threonine kinase domain of these effectors is distantly related to eukaryotic regulatory kinases; moreover, functional studies have suggested that these effectors can perturb the NF-κB pathway, interfere with innate immune responses, and inhibit apoptosis in infected host cells (26–28).

**FIG 5:**
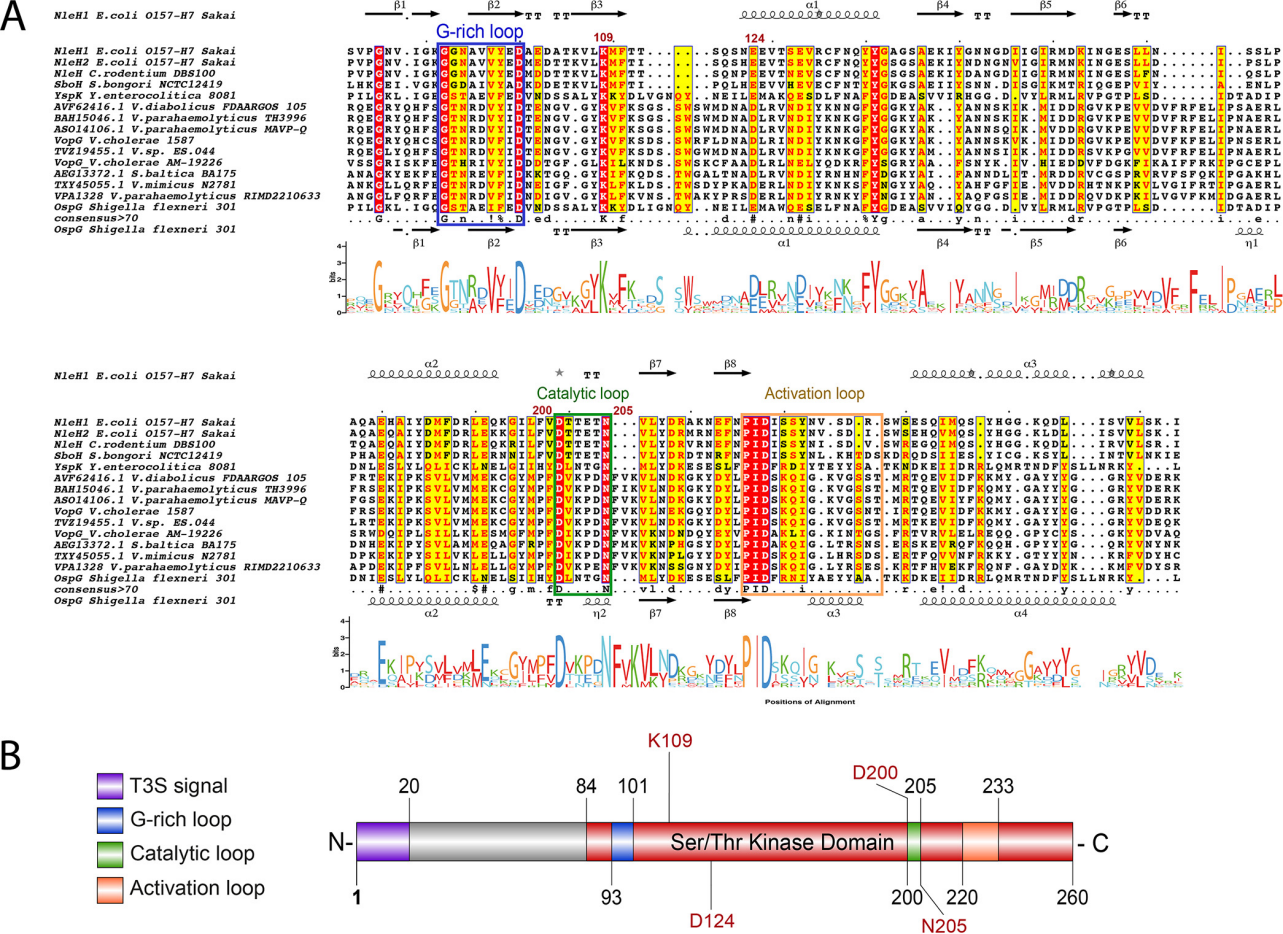
Conservation of the C-terminal domains of VopG homologs. (A) Multiple sequence alignment and WebLogo analysis of the C-terminal domains of VopG proteins (amino acids 84 to 260 in VPA1328) with the serine/threonine kinase domains of NleH proteins. The G-rich loop and catalytic and activation loops of the kinase are highlighted in colored boxes matching the schematic diagram in panel B. BLASTp alignment was performed using T-Coffee Expresso and visualized by ESPript 3.0. Amino acids within a red background correspond to positions with 100% identity; amino acids with a yellow background correspond to positions with >70% identity. The secondary structure of NleH1 and OspG is shown flanking the alignment (α, alpha helices; β, beta sheets; T, turns). (B) Schematic representation of VPA1328 highlighting the presence of the T3S signal as well as conserved regions and key catalytic residues of the putative serine/threonine kinase domain identified in panel A.

**FIG 6:**
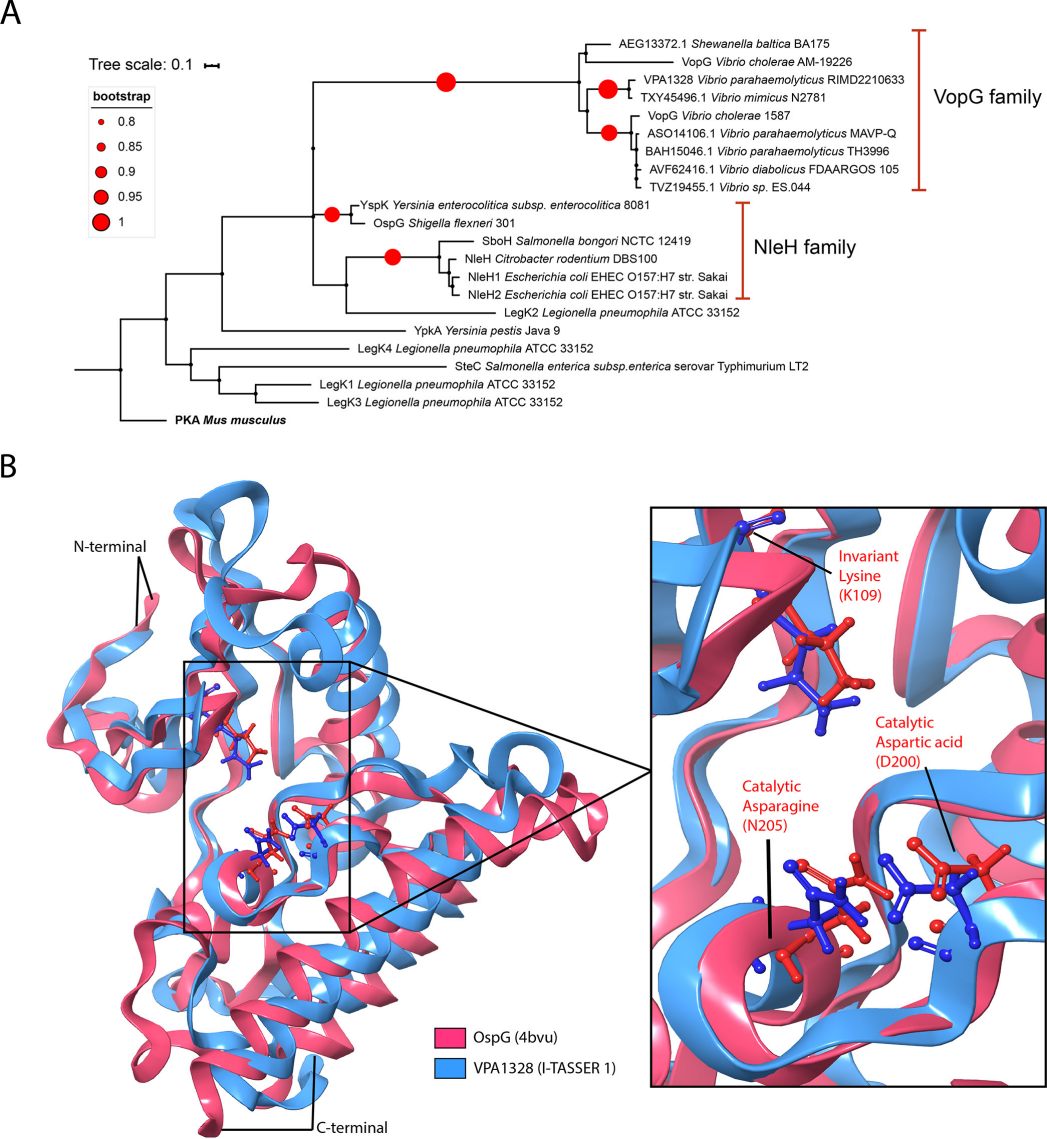
VopG homologs contain a NleH-like serine/threonine kinase domain. (A) Phylogenetic analysis of bacterial serine/threonine kinases. The analysis was performed with MEGA and visualized by iTOL. PKA, protein kinase A. (B) Comparative homology model of the C-terminal domain of VPA1328 (amino acids 84 to 260) superimposed on the known structure OspG (PDB accession no. 4BVU). The inset depicts the catalytic domain of OspG and the superimposed predicted structure of this region in VPA1328. Homology modeling was performed using the I-TASSER pipeline and visualized with MAESTRO.

Multiple sequence alignment of representatives of the NleH and VopG family of T3SS effector proteins were carried out to gain further insight into their similarity. Representatives from each clade of VopG proteins were included in these analyses. The analysis showed that the greatest similarity between VopG and NleH proteins is found in their C termini in the region that includes the characterized NleH serine/threonine kinase domain; in contrast, their N termini differ in both length and sequence (Fig. 5A and Fig. S4). VopG proteins contain all the critical amino acid residues and motifs important for kinase activity, including the conserved catalytic residues glycine of the G-rich loop, the aspartic acid (D) and the asparagine (N) of the catalytic loop (alignment positions 200 to 205 in VPA1328) and the PID motif of the activation loop (alignment positions 220 to 233 in VPA1328). In addition, VopG proteins also share the invariant lysine (alignment position 109) involved in the autophosphorylation of the NleH family, and which has been used as a proxy to measure kinase activity (27) (Fig. 5A and B). Thus, VopG family effector proteins harbor a NleH-like C-terminal serine/threonine kinase domain. Phylogenetic analysis of bacterial serine/threonine kinase domains also revealed the similarity of the kinase domains of VopG and NleH proteins (Fig. 6A). The VopG proteins clustered closer to the NleH proteins on this tree than to non-NleH serine threonine kinases from Legionella pneumophila, Yersinia pestis, and Salmonella enterica.

10.1128/mSphere.00599-21.4 FIG S4 Multiple sequence alignment of the amino acid sequences of the VopG homologs from V. parahaemolyticus RIMD2210633 and V. cholerae AM-19226 with the NleH1 and NleH2 proteins of E. coli O157:H7 and OspG protein of Shigella sonnei strain Ss046. BLASTp alignment was performed using M-Coffee and visualized by ESPript 3.0. Amino acids on a red background corresponds to positions with 100% identity, and amino acids on a yellow background correspond to positions with over 70% of identity. Download FIG S4, PDF file, 1.1 MB. Copyright © 2021 Plaza et al. 2021 Plaza et al. https://creativecommons.org/licenses/by/4.0/ This content is distributed under the terms of the Creative Commons Attribution 4.0 International license.

We derived a three-dimensional (3D) structural model of the C-terminal domain of VPA1328 using comparative homology modeling with I-TASSER (44) to gain further insight into the serine/threonine kinase domain of VopG proteins. In accord with the HHPred and pGenTHREADER analyses, I-TASSER identified NleH1, NleH2, and OspG as suitable models for comparative homology models using the crystal structures available for these proteins. Five models were obtained, and I-TASSER model 1 was chosen based on its error estimation, template modeling score (TM-score) and root mean square deviation (RMSD) values (Fig. S5). As shown in Fig. 6B, this model revealed the remarkable similarity of the predicted structure of the VPA1328 kinase domain with the NleH kinase domain. The structure of the catalytic pocket, including the positions of the predicted catalytic amino acid side chains (K109, D201, and N205) in VPA1328 and OspG structure overlap (Fig. 6B), strongly supporting the notion that the VopG family of proteins encode a NleH-like serine/threonine kinase domain.

10.1128/mSphere.00599-21.5 FIG S5 Estimated accuracy of the five protein structures of the C-terminal (amino acids 84 to 260) domain of VPA1328 obtained through comparative homology modeling using the I-TASSER pipeline. The locations of the predicted G-rich loop and catalytic and activation loops are highlighted. Download FIG S5, PDF file, 0.2 MB. Copyright © 2021 Plaza et al. 2021 Plaza et al. https://creativecommons.org/licenses/by/4.0/ This content is distributed under the terms of the Creative Commons Attribution 4.0 International license.

### VopG does not modulate T3SS2-mediated cytotoxicity or inhibit IL-8 production.

**FIG 7:**
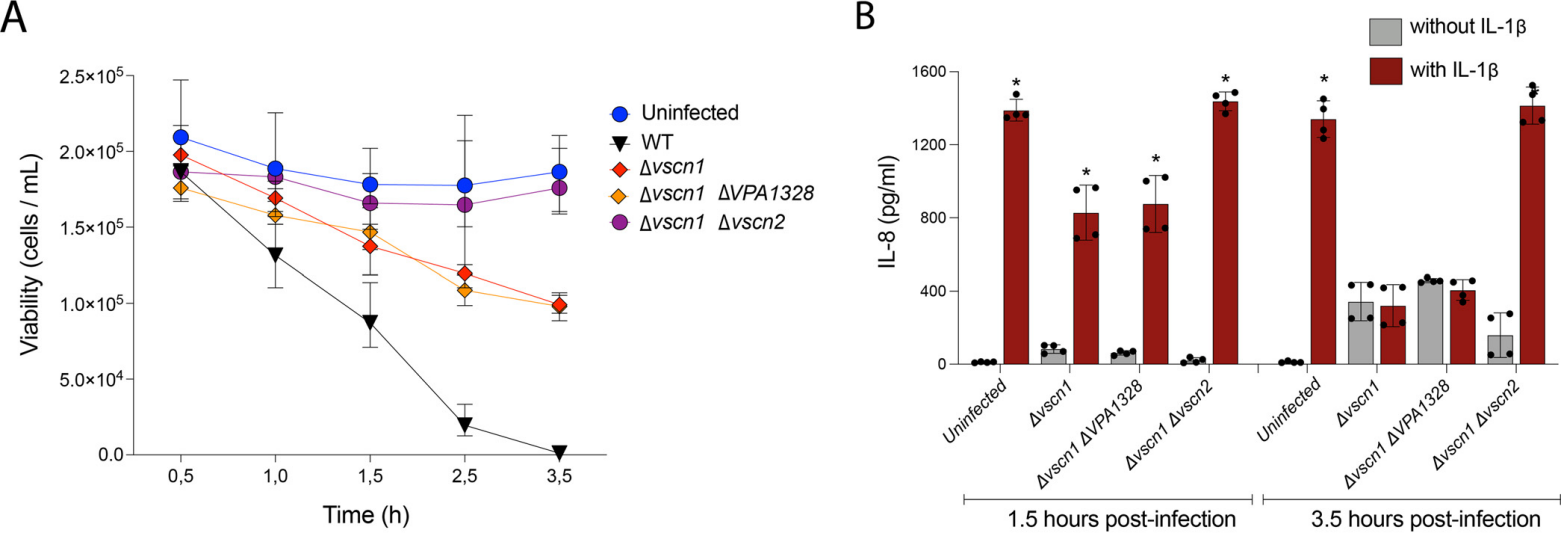
VopG does not modulate T3SS2-dependent cytotoxicity or suppression of IL-8 production. (A) Survival kinetics of Caco-2 cells infected with WT V. parahaemolyticus or derivatives with inactive T3SS1 (Δ*vscn1*), inactive T3SS1 without *vopG* (Δ*vscn1*, Δ*vopG*), or inactive T3SS1 and T3SS2 (Δ*vscn1*, Δ*vscn2*). Cell viability was evaluated by trypan blue exclusion tests at the indicated times. (B) Levels of IL-8 secretion (in picograms per milliliter) from HeLa cells infected with the indicated V. parahaemolyticus strains in the presence or absence of IL-1β (25 ng/ml) as an inducer. Error bars indicate standard deviations from two biological replicates each with two technical replicates. Asterisks indicate significant differences (multiple paired *t* test, *P* < 0.001) in comparison to the respective infected cells without IL-1β induction.

NleH family effector proteins inhibit IL-8 expression (26) and host cell death during infection (26–28). Since V. parahaemolyticus capacity to suppress IL-8 secretion and to induce host cell death is partially dependent on a functional T3SS2 (19, 45), we evaluated whether VopG contributes to these processes. A V. parahaemolyticus mutant strain harboring a deletion of *vopG* in the Δ*vscn1* genetic background was constructed to assess whether *vopG* modulates T3SS2-dependent killing of intestinal Caco-2 cells. As expected, Caco-2 cells were killed (<50% survival within 3.5 h of infection) by a V. parahaemolyticus strain harboring a functional T3SS2 (Δ*vscn1*, T3SS2+), whereas cells infected with a V. parahaemolyticus strain lacking both T3SSs (Δ*vscn1* Δ*vscn2*) were not (Fig. 7A). However, the absence of *vopG* (Δ*vscn1* Δ*vopG*, T3SS2+) did not alter survival of host cells infected with a functional T3SS2 (overlap of red and orange survival curves in Fig. 7A), suggesting that *vopG* does not modulate T3SS2-dependent cytotoxicity.

We then tested whether VopG contributes to T3SS2-dependent suppression of IL-8 production in infected cells. HeLa cells were infected with V. parahaemolyticus strains for 1.5 or 3.5 h and then IL-8 secretion was stimulated by incubating the cells with IL-1β for 90 min, as previously described (19). Infection by V. parahaemolyticus inhibited IL-8 secretion in a T3SS2-dependent fashion, but the absence of *vopG* did not influence this phenotype (Fig. 7B). Together, these data suggest that VopG does not modulate T3SS2-dependent host cell death or inhibit IL-8 production in infected cells.

## DISCUSSION

While all Vibrio parahaemolyticus strains harbor T3SS1, a hallmark of the pandemic V. parahaemolyticus O3:K6 clone and most human clinical V. parahaemolyticus isolates, is the presence of a second and phylogenetically distinct T3SS2. The latter T3SS is essential for both intestinal colonization and virulence in some animal models of disease (10, 12). Here, we found that a T3SS2 ORF (VPA1328) likely corresponds to a novel V. parahaemolyticus T3SS2 effector protein. This ORF, which is secreted in a T3SS2-dependent fashion, bears similarity to the VopG effector found in the V. cholerae AM-19226 T3SS2. The function of the latter VopG protein is unknown, but it has been shown to be translocated to host cells and linked to V. cholerae AM-19226’s pathogenicity. Bioinformatic analyses uncovered 123 nonredundant VopG-like proteins encoded in all three phylotypes of T3SS2 clusters in diverse *Vibrio* species and in Shewanella baltica. Interestingly, the evolutionary history of the T3SS2 phylotypes does not appear to correspond with the evolution of the three clades of VopG proteins that were uncovered by phylogenetic analysis. We found that the highly conserved C-terminal domains of VopG proteins bear striking structural similarity to the serine/threonine kinase domain of the NleH family of effectors found in enteric pathogens such as EHEC and *Shigella* (OspG). Thus, our findings support the idea that VopG effectors function as serine/threonine kinases in host cells.

The V. cholerae AM-19226 effector VopG had been classified as a V. cholerae-specific T3SS effector (29, 46), but our analyses showed that VopG homologs belong to a larger family of putative effector proteins that is widely distributed among *Vibrio* species, including V. parahaemolyticus, V. cholerae, V. mimicus, and V. diabolicus as well as in strains of S. baltica. Recently, Matsuda et al. (13) proposed classifying T3SS2 effectors proteins as “core” effectors if they are conserved in both V. parahaemolyticus and non‐O1/non‐O139 V. cholerae and as “accessory” effectors if they are not. According to this classification, our work suggests that VopG corresponds to a core effector protein due to its presence in multiple *Vibrio* species. However, VopG homologs are not present in the T3SS2 gene clusters identified in all *Vibrio* species; e.g., the T3SS2 cluster in Vibrio anguillarum (32) lacks a VopG homolog and not all clusters in *V*. *mimicus* (47) encode a recognizable VopG.

T3SS2 gene clusters are classified into three phylotypes (T3SS2α, T3SS2β, and T3SS2γ) that are believed to have been acquired through horizontal gene transfer events (13, 15, 47). Even though VopG homologs are not universally found in all T3SS2 gene clusters, we identified VopG homologs in all three T3SS2 phylotypes. Phylogenetic analysis identified three distinct VopG clades (Fig. 3C). These VopG clades did not correlate with T3SS2 phylotypes, i.e., all three clades were found in each T3SS2 phylotype. The apparent independent evolution of T3SS2 phylotypes and VopG clades supports the possibility that *vopG* genes have been independently acquired by different T3SS2 lineages.

The absence of *vopG* genes from certain T3SS2 clusters could be explained either by loss of *vopG* loci due to deletion event(s) or independent acquisition of *vopG* in some T3SS2 clusters. The presence of a second *vopG* homolog flanked by IS elements in V. parahaemolyticus strain FORC014 suggests insertion sequences may play a role in mobilizing *vopG* genes. These sequences bear similarity to the ISVpa3 insertion sequence first reported in V. parahaemolyticus RIMD2210633 (33). Since insertion sequences have been shown to shape bacterial genomic islands through rearrangements, insertion, and deletion events, it is plausible that ISVpa3-like elements have shaped the evolution of T3SS2 gene clusters through similar mechanisms. The apparent mobility of *vopG* loci adds an additional layer of complexity to our understanding of T3SS2 clusters. That is, these clusters appear to have been spread via horizontal gene transfer events among marine bacteria, and their repertoire of effector proteins appears to be “tunable” through independent horizontal gene transfer or rearrangement events.

Analysis of the amino acid sequences of the 123 nonredundant VopG homologs identified here revealed a particularly high degree of conservation in their C termini. This region of VopG proteins was found to be very similar to the conserved serine/threonine kinase domain in the NleH family of T3SS effector proteins. Thus, the conservation of this part of VopG effectors is likely explained by the presence of a functional kinase domain. Structural predictions, which showed that VopG proteins contain all the residues that constitute that catalytic pocket of NleH proteins, strongly support this hypothesis.

The NleH family of effectors contain a eukaryotic-like serine/threonine kinase domain that independently evolved in bacteria (37, 38). VopG homologs harbor each of the key residues described in the NleH family of protein kinases. The classification of the NleH proteins as a distinct bacterial kinase family was made through structure-based phylogenetic analysis (37). Phylogenetic analysis showed that the C termini of VopG proteins have more similarity to the kinase domain of NleH proteins than to other bacterial protein kinases (Fig. 6A), but further structural information is required to determine whether VopG proteins are novel members of the NleH family or represent a distinct family on their own.

Although the conservation of key catalytic residues in VopG and NleH proteins provides evidence that supports the notion that VopG proteins are functional serine/threonine kinases, it is more problematic to speculate that their biological function is conserved as well. To date, NleH proteins have been shown to perturb the NF-κB pathway and impact cell survival and innate immune responses during infection through different molecular mechanisms (26–28, 40, 48). Both NleH1 and NleH2 proteins bind the host protein RPS3 leading to inhibition or activation of the NF-κB pathway, respectively (26, 49), but only NleH1 suppresses IL-8 expression during EHEC infection (26). Despite these differences in control of IL-8 expression, both NleH1 and NleH2 inhibit apoptosis through interaction with the Bax inhibitor 1 protein (27). The *Shigella* OspG protein inhibits the NF-κB pathway by inhibiting the proteasomal destruction of IκBα (42), and the SboH protein of Salmonella bongori blocks intrinsic apoptotic pathways (28). The V. parahaemolyticus T3SS2 causes cell death (18, 50, 51) and suppresses IL-8 secretion through perturbation of the NF-κB pathway (19, 45). While the exact mechanism of T3SS2-mediated host cell death is unknown, the VopZ effector protein plays an important role in inhibiting IL-8 production (19). Our tissue culture-based infection experiments did not reveal that VopG modulates T3SS2-dependent host cell death or IL-8 suppression (Fig. 7). Two possible scenarios might explain these observations. (i) VopG’s contribution to these phenotypes are masked by the redundant effects of additional effectors such as VopZ. (ii) VopG targets host pathways that do not influence cell death or IL-8 synthesis. The N-terminal region of NleH proteins has been linked to substrate recognition and the observed functional differences between the NleH1 and NleH2 proteins (26, 37, 40). In this context, it is tempting to speculate that the sequence divergence observed within the N-terminal region of VopG homologs (see Fig. S3 and S4 in the supplemental material) has functional implications.

In summary, our work identifies a new family of VopG proteins that are likely T3SS2 effectors. These proteins contain a distinctive NleH-like serine/threonine kinase domain. Future biochemical and structural studies are required to corroborate these predictions. Moreover, defining the role(s) of these effectors in the pathogenicity and/or environmental adaptation of the diverse *Vibrio* and *Shewanella* species that encode them will be fruitful.

## MATERIALS AND METHODS

### Bacterial strains and growth conditions.

All bacterial strains and plasmids used in this study are listed in Table S1 in the supplemental material. V. parahaemolyticus RIMD2210633 (3) and its Δ*vscn1*, Δ*vscn2*, and Δ*vscn1* Δ*vscn2* derivatives (19) were used in this study. Bacterial strains were routinely cultured in LB medium or on LB agar plates at 37°C. Culture medium was supplemented with the following antibiotics and chemicals: 0.04% bovine and ovine bile (Sigma catalog no. B8381); 5 μg/ml and 20 μg/ml chloramphenicol for V. parahaemolyticus and E. coli strains, respectively; 1 mM isopropyl-β-d-thiogalactopyranoside (IPTG) to induce expression vector pCyaA in secretion and translocation assays.

10.1128/mSphere.00599-21.6 TABLE S1 Bacterial strains and plasmids used in this study. Download Table S1, XLSX file, 0.01 MB. Copyright © 2021 Plaza et al. 2021 Plaza et al. https://creativecommons.org/licenses/by/4.0/ This content is distributed under the terms of the Creative Commons Attribution 4.0 International license.

### Eukaryotic cell culture and maintenance conditions.

Caco-2 cells (ATCC HTB-37) were maintained in Dulbecco’s modified Eagle medium (DMEM) (Gibco) supplemented with 10% fetal bovine serum (FBS) (Gibco) (DMEM−15% FBS) at 37°C in 5% CO_2_. Cells were grown at 37°C with 5% CO_2_ and routinely passaged at 70 to 80% confluence.

### T3SS2 secretion assays.

A reporter fusion was constructed between VPA1328 and the CyaA reporter encoded in plasmid pCyaA (a pMMB207 derivative) (19), generating plasmid pVPA1328 (VopG-CyaA), to investigate VPA1328 (VopG) secretion. A VopV-CyaA fusion (pVopV-CyaA) (19) was used as a positive control for T3SS2-dependent secretion, and the empty plasmid pCyaA was used as a negative control. Each plasmid was transformed into V. parahaemolyticus by electroporation as previously described (19). Secretion assays were performed by growing each V. parahaemolyticus strain for 1.5 h in LB medium supplemented with 0.04% bile. When cultures reached an optical density at 600 nm (OD_600_) of 0.5 to 0.6, 1 mM IPTG was added to induce expression of the CyaA reporter fusion protein. After 1.5 h of induction, culture supernatants were collected by two centrifugations, one at 5,000 rpm for 20 min and a final centrifugation at 13,000 rpm for 5 min. The supernatants were then filter sterilized through a 0.22-μm filter and concentrated 100-fold by repeated centrifugation at 5,000 rpm for 30 min with an Amicon Ultra-15 centrifugal filter unit (Millipore) with a 10-kDa molecular weight cutoff and with two washes with 15 ml of 1× phosphate-buffered saline (PBS) as an exchange buffer. Prior to concentrating the culture supernatant, bovine serum albumin (BSA) (1 mg/ml) was added to serve as a concentration/loading control. Whole-cell lysates were prepared by solubilizing the bacterial pellets in 1× Laemmli buffer. Lysate and supernatant samples were processed for sodium dodecyl sulfate-polyacrylamide gel electrophoresis (SDS-PAGE) analysis by mixing them with loading buffer and boiling for 5 min; they were then run on 4 to 20% Mini-PROTEAN TGX Precast Gels (Bio-Rad) per 30 min at 200 V. For immunoblot analysis, gels were transferred to iBlot2 transfer stacks of polyvinylidene difluoride (PVDF) membranes (Invitrogen) and blocked by EveryBlot blocking buffer (Bio-Rad catalog no. 12010020). The Pierce Coomassie Plus (Bradford) assay kit (Thermo Fisher catalog no. 23236) was used for determination of protein concentrations. Antibodies were used at the following dilutions: anti-CyaA (mouse monoclonal, 1:2,000; Santa Cruz Biotechnology catalog no. sc-13582), anti-RpoB (rabbit monoclonal, 1:2,000; Abcam catalog no. ab191598), anti-mouse IgG conjugated to horseradish peroxidase (HRP) (anti-mouse IgG-HRP) (goat polyclonal 1:10,000; Thermo catalog no. 62-6520), and anti-rabbit IgG (H+L) secondary antibody conjugated to HRP (goat polyclonal 1:10,000; Invitrogen catalog no. G21234). The blots were developed with SuperSignal West Pico Plus substrate (Thermo Fisher catalog no. 35060), and imaging was performed on a C-DiGit Blot Scanner (LI-COR Biosciences). All blots are representative of at least three biological replicates.

### Translocation of effector-CyaA fusion proteins.

CyaA reporter fusion-based protein translocation assays were performed as previously described (18). Briefly, Caco-2 cells were seeded at 1.5 × 10^4^ cells/well and cultured in DMEM−10% FBS for 2 days in a 96-well plate at 37°C in 5% CO_2_. V. parahaemolyticus RIMD2210633 Δ*vscn1* and Δ*vscn1* Δ*vscn2* strains containing pCyaA, pVopV-CyaA or pVopG-CyaA were grown for 1.5 to 2 h until they reached an OD_600_ of 0.6 in LB medium supplemented with 0.04% bile. The infection assay in Caco-2 cells was performed for 1 h at 37°C and 5% CO_2_ and at a multiplicity of infection (MOI) of 50. The intracellular cyclic AMP (cAMP) levels in Caco-2 cells were determined using cAMP Biotrak enzyme immunoassay (EIA) kit (Cytiva catalog no. RPN2251) as described previously (52). Statistical analysis was performed with GraphPad Prism version 9 (GraphPad Software, San Diego, California, USA).

### T3SS2-dependent cell death and IL-8 secretion assays.

For cell survival assays, Caco-2 cells were seeded at 8.0 × 10^4^ cells/well into six-well plates and grown for 2 days in complete media. V. parahaemolyticus strains were cultured overnight and the next day diluted 1:100 into LB liquid media containing 0.04% bile (to induce T3SS2 expression) and grown for 2 h until attaining an OD_600_ of 0.6. Cells were infected at an MOI of 1 and incubated at 37°C with 5% CO_2_. At each time point assayed (0.5, 1.0, 1.5, 2.5, and 3.5 h.), the medium was replaced with fresh complete DMEM medium supplemented with 100 μg/ml of gentamicin. Cells were incubated overnight, and surviving cells were quantified either by trypan blue exclusion (0.4% trypan blue) and counted on a hemocytometer (Neubauer cell chamber). For the detection of secreted IL-8, Caco-2 cells were seeded at 1.0 × 10^5^ cells/well into 12-well plates and cultured in DMEM−10% FBS for 24 h at 37°C in 5% CO_2_. V. parahaemolyticus strains were cultured, and T3SS2 was induced as described above. Cells were infected at an MOI of 1 and incubated at 37°C with 5% CO_2_ for 1.5 or 3.5 h, and then, the infection was terminated by addition of gentamicin (100 μg/ml). In parallel with gentamicin, the cells were treated with IL-1β (25 ng/ml) or left untreated for 90 min. IL-8 in culture supernatant was then measured using a human IL-8 ELISA kit (ab46032). Statistical analysis was performed with GraphPad Prism version 9 (GraphPad Software, San Diego, California, USA).

### Sequence and phylogenetic analysis.

Identification of VopG orthologs was carried out using the VPA1328 amino acid and nucleotide sequences as queries in BLASTp, BLASTn, BLASTx, tBLASTn, and tBLASTx analyses (64) using publicly available bacterial genome sequences of the NCBI database (December 2020). A 94% sequence length, 40% identity and 60% sequence coverage threshold were used to select positive matches. Sequence conservation was analyzed by multiple sequence alignments using MAFFT (53) and T-Coffee Expresso (54) and visualized by ESPript 3.0 (55). WebLogo analysis was performed using multiple sequence alignments (56). Comparative genomic analysis of the T3SS2 gene clusters was performed using the multiple aligner Mauve (57) and the IslandViewer 4 pipeline (58) and EasyFig v2.2.2 (65). Nucleotide sequences were analyzed by the sequence visualization and annotation tool Artemis version 18.1 (59). Multiple sequence alignments were used for phylogenetic analyses that were performed with the Molecular Evolutionary Genetics Analysis (MEGA) software version 7.0 (60) and visualized by iTOL (61). Phylogenetic trees were built from the alignments by the bootstrap test of phylogeny (2000 replications) using the maximum-likelihood (ML) method with a Jones-Taylor-Thornton (JTT) correction model.

### Remote homology prediction and homology modeling.

Remote homology prediction of VPA1328 was performed using HHpred (35) and pGENTHREADER (36) on the PSIPRED server (62). Protein structure models of the VPA1328 C-terminal domain were obtained using I-TASSER (44), a protein structure homology-modeling server. Protein structure visualization and template alignment and superposition were performed using MAESTRO (63).
