# Supplementary figures and images for: Identification of a Family of Vibrio Type III Secretion System Effectors That Contain a Conserved Serine/Threonine Kinase Domain
Source: mSphere. 2021 Aug 4;6(4):e00599-21. doi: 10.1128/mSphere.00599-21 (PMC8386410; doi:10.1128/mSphere.00599-21)

A

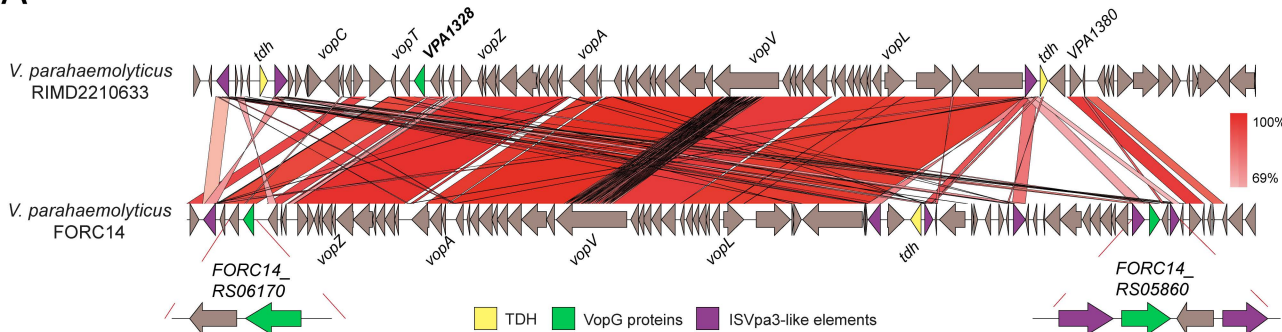

B

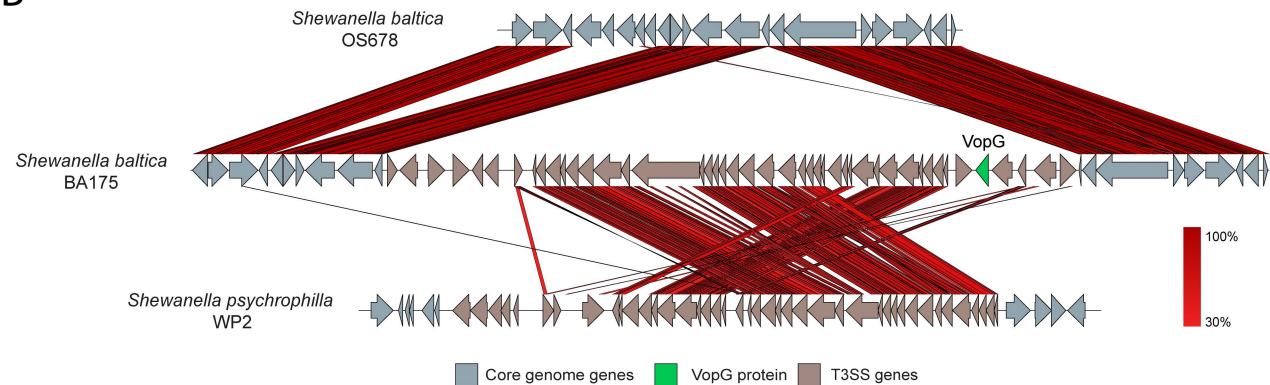

Supplement: FIG S1 [file msphere.00599-21-sf001.pdf]

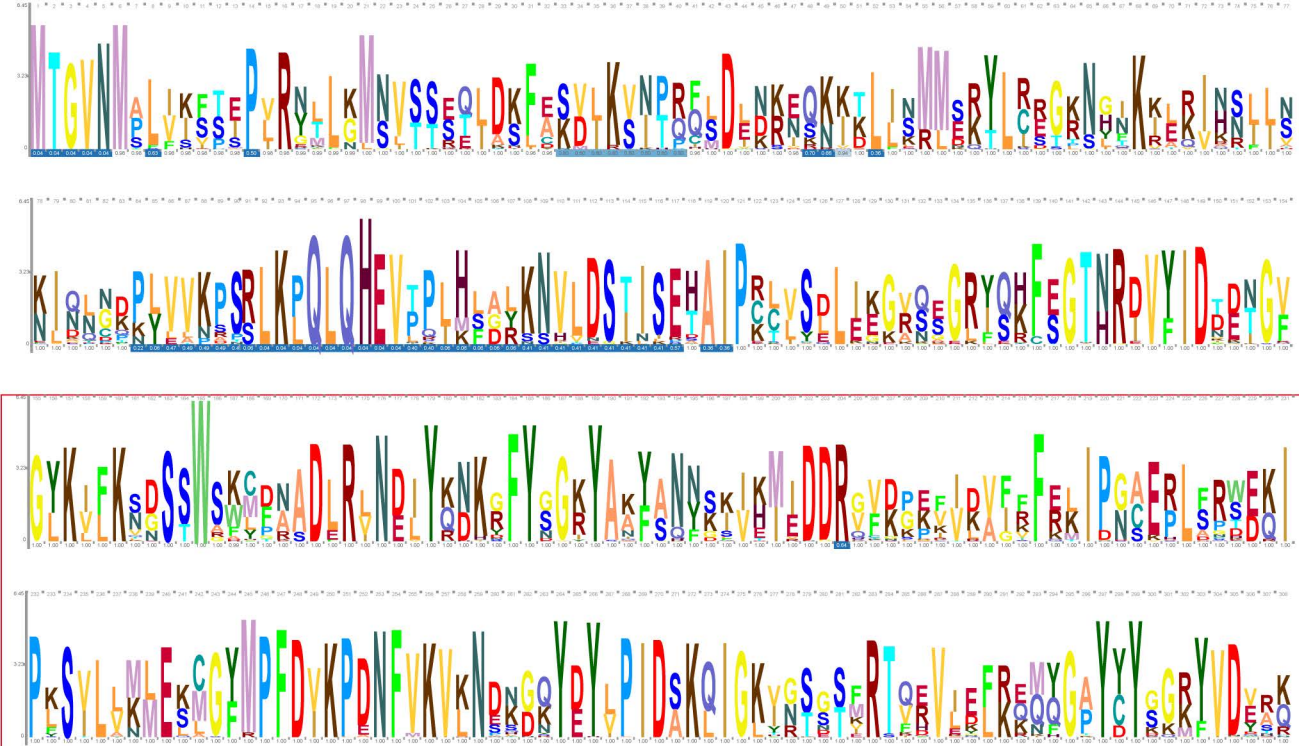

C-terminal region

Supplement: FIG S3 [file msphere.00599-21-sf003.pdf]

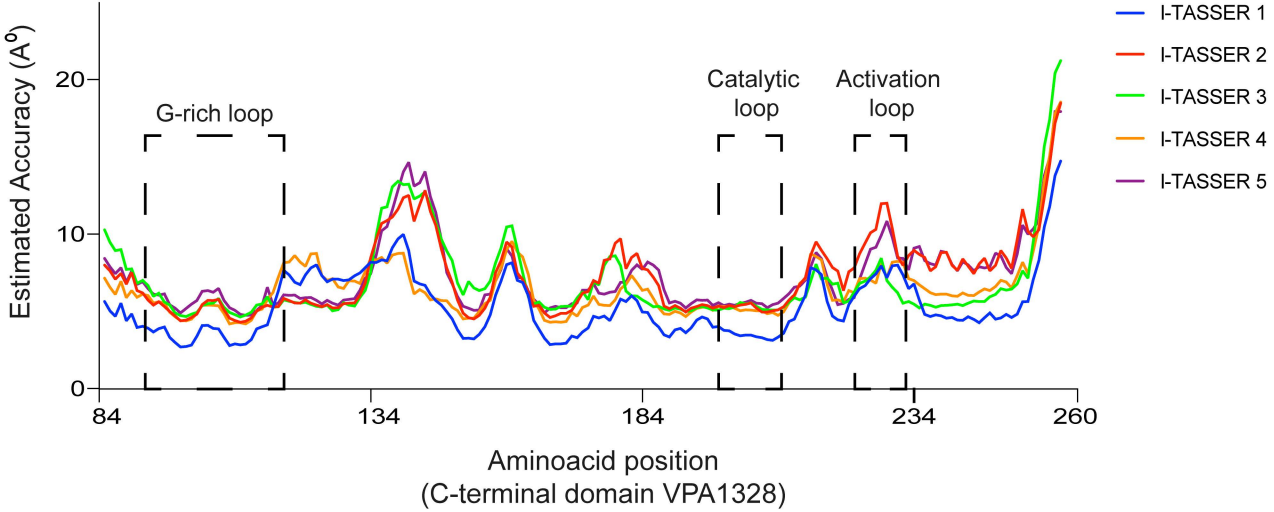

Supplement: FIG S5 [file msphere.00599-21-sf005.pdf]
