# Supplementary material for: Identification of a Family of Vibrio Type III Secretion System Effectors That Contain a Conserved Serine/Threonine Kinase Domain
Source: mSphere. 2021 Aug 4;6(4):e00599-21. doi: 10.1128/mSphere.00599-21 (PMC8386410; doi:10.1128/mSphere.00599-21)
Supplement: FIG S2 [file msphere.00599-21-sf002.pdf]

```
                1        10        20        30        40        50        60        70        80        90
FORC14_RS06170  ATGTCTTTGTTTCTGTACCATCTCCTATTAGAGGGATGGTAAAAATGAATTTATCATCTCAAGAACTAGCTAAGCTTTGTTGTATGGAT
FORC14_RS05860  ATGTCTTTAATCCATATTTCCTCTCCTATTCGTAGAATGTTAAACATGAATATCTCATCTCGAGAGTTAGCTGATATCTATCACATGGAT
consensus>70    ATGTCTTT..T...T.T..C.TCTCCTATT.G..G.ATG.TAAA.ATGAAT.T.TCATCTC.AGA..TAGCT.A..T.T.T...ATGGAT

                         100       110       120       130       140       150       160       170       180
FORC14_RS06170  ATAAAGAAAATCTCCAATAAAGATATTAAACGTCTAGATAGGACTTTAATTTCGACTACAAACTTGTTTAAGAGAGCGCAAGTTAAGCGA
FORC14_RS05860  ATTAAAAGCATATCCAATAAAGATATTGAACGTTTAAATAAAACCTTGAAATCGACAAAAAACTTATTTAAGAGAACGAAAGTACAACGA
consensus>70    AT.AA.A..AT.TCCAATAAAGATATT.AACGT.TA.ATA..AC.TT.A..TCGAC.A.AAACTT.TTTAAGAGA.CG.AAGT..A.CGA

                         190       200       210       220       230       240       250       260       270
FORC14_RS06170  CTTTTGGTTAAACTTGACCAATGTCCACCCGAGCATCCTAAAACGGTTTATGAGTTAATTGGTAAAGCCAATGGAGGGTTGTTCAGCGT
FORC14_RS05860  CTTCTAACTAAAATTGGTTACTGTCAACCAGAACACCCCAAAAGTGTTTGCGAGCTAATTAATAAAGCAAACAGAGGATTACTTCAGCGT
consensus>70    CTT.T...TAAA.TTG...A.TGTC.ACC.GA.CA.CC.AAAA..GTTT..GAG.TAATT..TAAAGC.AA..GAGG.TT..T.CAGCGT

                         280       290       300       310       320       330       340       350       360
FORC14_RS06170  TTTGAAGGAACTAACAGAGACGTATTTATTGATAACGATATTGGTGTTGGATATAAACTATTTAAAGTAAATAGTACATGGGCTAAGTTT
FORC14_RS05860  TTCGAAGGGACGAATCGAGATGTCTTTATTGATAATGAAATTGGATTTGGTTATAAACTATTTAAATTGGATAGCACATGGTCAGATCAC
consensus>70    TT.GAAGG.AC.AA..GAGA.GT.TTTATTGATAA.GA.ATTGG..TTGG.TATAAACTATTTAAA.T..ATAG.ACATGG.C..A....

                         370       380       390       400       410       420       430       440       450
FORC14_RS06170  CCAAGATCCGATGAGCGTCTTAATGATATTTATAGGAATAAATATTTTTATAATGGTAAATATGCTAATTATGCTCAGTTTGATTCTATA
FORC14_RS05860  CCCAAGGCCGATGAAAGATTGAACGATATTTATAAAAACAAAAGTTTTTATAATGGCTATTATGCTCAATATGCTCATTTTGGTTTTATA
consensus>70    CC.A...CCGATGA..G..T.AA.GATATTTATA..AA.AAA..TTTTTATAATGG..A.TATGCT.A.TATGCTCA.TTTG.TT.TATA

                         460       470       480       490       500       510       520       530       540
FORC14_RS06170  GAGATGATTGACGATCGTCAAGTTGACAAGCCTAAAATATTAGTCGGCGTTTTTAAGATGATCGACGGAGCTGAGCGTTTGGCGCCATAC
FORC14_RS05860  GAAATGGTCGATGATCGTCATACTAATAAACCTAAAGTATTAGTTGGAATATTTAGAACAATACCAGGTGCTGAACGTTTAGATCCTAAA
consensus>70    GA.ATG.T.GA.GATCGTCA...T.A.AA.CCTAAA.TATTAGT.GG..T.TTTA..A..AT....GG.GCTGA.CGTTT.G..CC..A.

                         550       560       570       580       590       600       610       620       630
FORC14_RS06170  GAAAAAATCCCATTCTCAGTTTTACTAAATTTAGAATTGTTAGGTTATATGCCTTTTGATGTGAAGCCTGATAATTTTGTCAAAGTAAAA
FORC14_RS05860  GAAAAAATACCATATTCTATTTTAGTAAAGTTGGAACTATTAGGTTATATGCCGTTTGACATTAAGCCTGACAATTTTGTCAAGGTAAAA
consensus>70    GAAAAAAT.CCAT..TC..TTTTA.TAAA.TT.GAA.T.TTAGGTTATATGCC.TTTGA..T.AAGCCTGA.AATTTTGTCAA.GTAAAA

                         640       650       660       670       680       690       700       710       720
FORC14_RS06170  AATTCTGCTGGAAATTATGACTACCTTCCTATTGACTCTAAGCAAATTGGTTTGTACAAAAGTGACTCTAAGAGAACATTTCATGTAGAT
FORC14_RS05860  AACCCTTTGGGTTATTATGATTACCTTCCTATTGATTCTAAACAAATAGGTTTGCATCGAAGTGATTCAGAAAGAACATTTCAAGTAATA
consensus>70    AA..CT...GG..ATTATGA.TACCTTCCTATTGA.TCTAA.CAAAT.GGTTTG.A...AAGTGA.TC..A.AGAACATTTCA.GTA...

                         730       740       750       760       770       780
FORC14_RS06170  AAATTTAGACGAAACTTCGGGGCTTATGATTATAAAAAGATGTTTGTCGATTATTCGCGTTAG
FORC14_RS05860  AATTTTAGAAAAAAATACGGAACTTATTATTATAATAAGAGATTTGTTGACTATCATTGTTAG
consensus>70    AA.TTTAGA..AAA.T.CGG..CTTAT.ATTATAA.AAGA..TTTGT.GA.TAT....GTTAG
```
