# Supplementary material for: Identification of a Family of Vibrio Type III Secretion System Effectors That Contain a Conserved Serine/Threonine Kinase Domain
Source: mSphere. 2021 Aug 4;6(4):e00599-21. doi: 10.1128/mSphere.00599-21 (PMC8386410; doi:10.1128/mSphere.00599-21)
Supplement: FIG S4 [file msphere.00599-21-sf004.pdf]

```
                1        10        20        30        40        50        60        70        80
NleH1_Q8X831    MLSPYSV.NLGCSWNSLTRNLTSPDNRVLSSVRDAAVHSDNGAQVKVGNRTYRVVATDNKFCVTRESHSGCFTNLLHRLGWPKGEISRKI
NleH2_Q8XAL6    MLSPSSI.NLGCSWNSLTRNLTSPDNRVLSSVRDAAVHSDSGTQVTVGNRTYRVVVTDNKFCVTRESHSGCFTNLLHRLGWPKGEISRKI
OspG_Q3YTH2     MK............................................................................I...........
VPA1328         MSLFSVPSPIRGMLNM..NLSSRELA...........................KLCCMDIKKIS........NKDIKRL...........
VopG_A33_1697   MPIISSI.PLRYTLGM..SVTTSTID...........................SF.AKDIKSITQQLDEDKNKKLISMM...........
consensus>70    M.........................................................n...v...............l...........

                90       100       110       120                 130       140       150       160
NleH1_Q8X831    EVMLNASPVSAAMERGIVHSNRPDLPPVDYAPPELPSVDY...............NRLSVPGNVIGK..GGNAVVYEDAEDATKVLKMFT
NleH2_Q8XAL6    EAMLNTSPVSTTIERGSVHSNRPDLPPVDYAQPELPPADYTQS..E..LPR.VSNNKSPVPGNVIGK..GGNAVVYEDMEDTTKVLKMFT
OspG_Q3YTH2     TS....TIIQTPFPFENNNSHA.GI.............VT.................EPILGKLIGQ..GSTAEIFEDVNDSSALYKKYD
VPA1328         DR....TLISTPSLFKRAQVKR.LLVKLDQCPPEHPKTVYELI..............GKANGGLFQRFEGTNRDVFIDN.DIGVGYKLFK
VopG_A33_1697   ER....YLCRGRNGIKKEKINN.ILSNINNGKPP..KNVLDSIISEHAIPCCLSLLEEGVSSGRISKFEGTHRIVYIDD.DTGFGLKILK
consensus>70    e.......v...............l...d...p..........................v.g..i....G....!%.D..D.....K.f.

                      170       180       190       200            210       220       230
NleH1_Q8X831    TSQS.N.......E...EVTSEVRCFNQYYGAGSAEKIYGNNGDIIGIRMD.....KINGESLLN..ISSLPAQAEHAIYDMFDRLEQKG
NleH2_Q8XAL6    ISQS.H.......E...EVTSEVRCFNQYYGSGSAEKIYNDNGNVIGIRMN.....KINGESLLD..IPSLPAQAEQAIYDMFDRLEKKG
OspG_Q3YTH2     LIGNQY......NEILEMAWQESELFNAFYGDEASV.VI.QYGGDVYLRML.....RVPGTPLSDIDTADIPDNIESLYLQLICKLNELS
VPA1328         VNST.WAKYPRSDERLNDIYRNKYFYNGIYANYAQFGSI......EMIDDRQVDKPKILVGVFKMIDGAERLAPDEKIPFSVLLNLELLG
VopG_A33_1697   NDSS.WSKCFAADLRLNELYQDKRFYSGRYAAFSNYKIV......HIEDDV.FDGKFIKAIFFRKIPGCEPLSRWDQIPLSILLKLESMG
consensus>70    .............e...e...#...%n..Y.......i..................!..................#.........L#..g

                     240       250       260       270       280       290
NleH1_Q8X831    ILFVDTTETNVLYDRAKNEF...NPIDISSY.NVSDRSWSES.QIMQ.SYHGGKQDLISVVLSK.I
NleH2_Q8XAL6    ILFVDTTETNVLYDRMRNEF...NPIDISSY.NVSDISWSEH.QVMQ.SYHGGKLDLISVVLSK.I
OspG_Q3YTH2     IIHYDLNTGNMLYDKESESL...FPIDFRNIYAEYYAATKKDKEIIDRRLQMRTNDFYSLLNRKYL
VPA1328         YMPFDVKPENFVKVKNSSGNYDYIPIDSKQI.GLYRSESKRTFHVEKFRQNFGAYDYKKMFVDYSR
VopG_A33_1697   FMPFDIKPDNFVKVKNDNDQYEYVPIDAKLI.GKINTGSFRTVRVLELREQQGPYCYSGKYVDVAQ
consensus>70    .l..D....N.......n......PID.................!.q.....g..d..........
```
